# A BERTweet-based design for monitoring behaviour change based on five doors theory on coral bleaching campaign

**DOI:** 10.1186/s40537-022-00615-1

**Published:** 2022-05-31

**Authors:** Gabriela Nathania Harywanto, Juan Sebastian Veron, Derwin Suhartono

**Affiliations:** Computer Science Department, School of Computer Science, Bina Nusantara University, Jakarta, 11480 Indonesia

**Keywords:** Five Doors Theory, Behaviour change, Tweet classification, BERTweet model, Ensemble technique, Embedding extraction

## Abstract

Coral reefs are very important ecosystem which are the foundation of all life on this earth, but now they are under threat. Coral bleaching are happening now at a serious rate and the ultimate goal of conservation effort toward this issue is behaviour change. One of the most important parts of conservation effort is monitoring. However, monitoring the success of the coral bleaching campaign on behaviour change requires extensive data collection so traditional methods are not effective because they require resources that may not be met. The goal of this study is to build fast and vast automation in analyzing the stage of behaviour change. Social media data has prospect to become good alternative to be used because social media usage is currently increasing every year, including Twitter. Therefore, an automatic classification model was designed which can identify the stages of behaviour change based on the Five Doors Theory on Twitter. Five Doors Theory define 5 stages of behavior change: Desirability, Enabling Context, Can Do, Buzz, and Invitation. The data was fetched through a trusted repository, Mendeley Data, with title "An Annotated Dataset for Identifying Behaviour Change Based on Five Doors Theory Under Coral Bleaching Phenomenon on Twitter". There are 1,222 tweets with keywords related to coral bleaching that have been annotated according to the behaviour change stages. There are two proposed designs: embedding extraction which utilizes the output of each encoder layer in BERTweet and stacking ensemble which uses several BERTweet models with different hyperparameters that are ensembled using a logistic regression model. The best accuracy of 0.7796 with an f1-score of 0.7945 was obtained in the stacking ensemble design scenario. The classification model created can identify each class at the stage of behaviour change well, even though the dataset is unbalanced in its distribution. The proposed design has a performance that exceeds all baseline models and the standalone BERTweet. In conclusion, the automatic classification model create the process of monitoring the stages of behavior change run effectively and efficiently so that the success of the coral bleaching campaign can be monitored and achieved.

## Introduction

Coral reefs support an extremely high level of biodiversity and provide an important ecosystem foundation for millions of people [1]. Directly, economic activities that depend on marine resources are strongly supported by the existence of coral reefs. Coral reefs experience various challenges: long-term changes in ocean and atmosphere interactions, rising sea temperatures, increasing CO_2_ levels, weather changes due to major storms, earthquakes, volcanic eruptions, and extreme weather changes [2]. Those challenges lead to the phenomenon of coral bleaching which is a threat to the biodiversity of coral reefs worldwide. Global coral bleaching in 2014–2017 was the third time in the last 20 years and killed thousands of square kilometers of coral reefs and other coral organisms [3, 4].

In natural way, coral bleaching can recover within a certain period of time. However, due to the continuous increase in seawater temperatures, the recovery capacity cannot compensate for the bleaching phenomenon that occurs. Awareness of this issue is very important as the first effort to conserve and maintain coral reefs. Raising awareness of the value of biodiversity, knowing how to conserve it, and using it sustainably is the key to success in achieving all of the *Aichi Biodiversity Targets* [5]. The target of increasing awareness is stated in Aichi Target 1. However, the success of this target is difficult to monitor and evaluate traditionally [6].

The use of social media has increased significantly over the last few years. Social media can be a prospective source of data to monitor and evaluate public awareness of environmental issues [7], including coral bleaching. The ultimate goal of efforts or campaigns on the issue of the coral bleaching is not only raising awareness, but also changes in the behaviour of the community so that they are actively involved in conservation efforts. According to Robinson, there are 5 stages of behaviour change called the Five Doors Theory, including: Desirability, Enabling Context, Can Do, Buzz, dan Invitation [8].

**Fig. 1:**
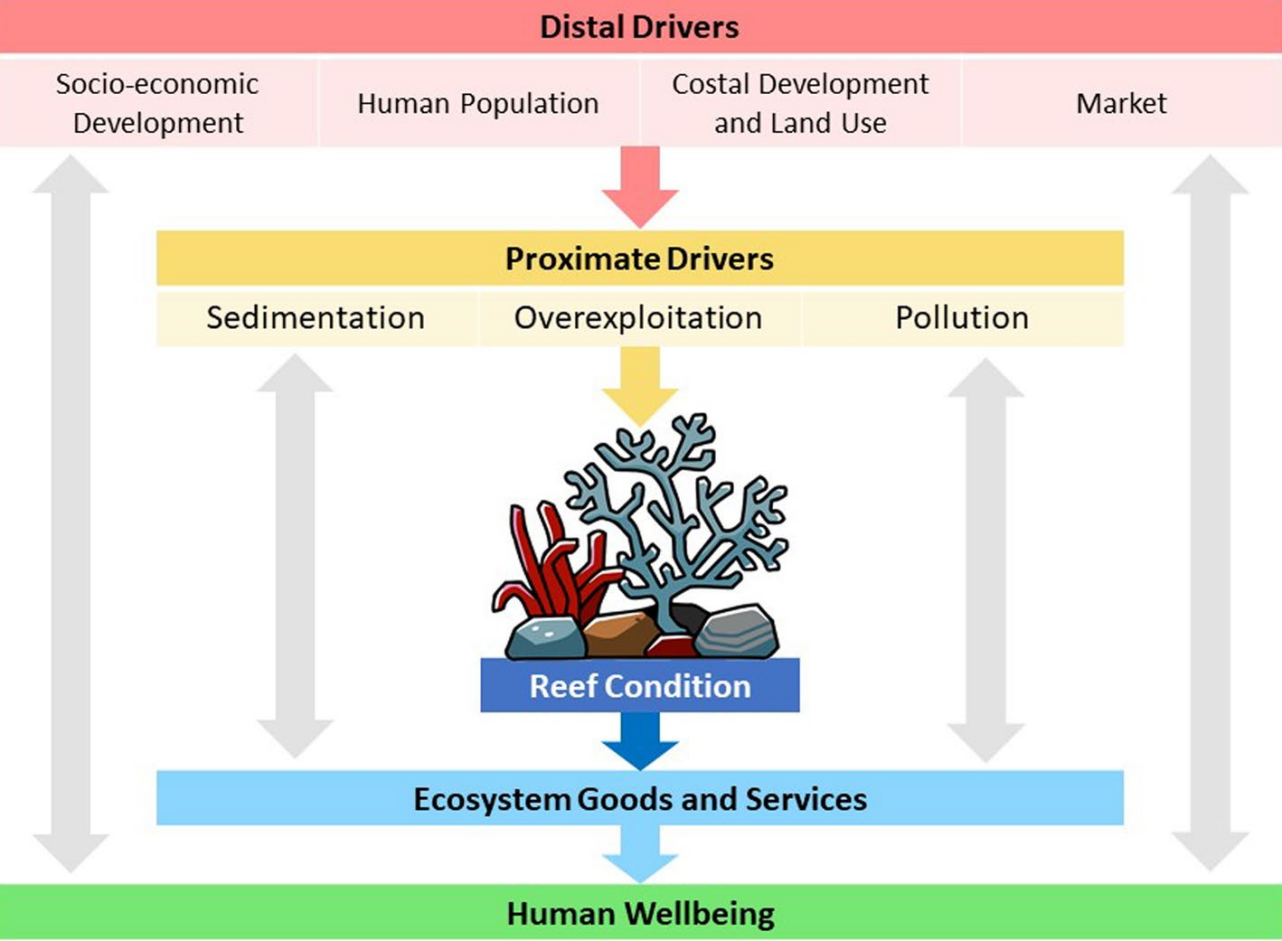
The interrelationships and reciprocity between humans and coral reefs

Through social media, the development of various studies and efforts related to coral bleaching issues that have taken place in various regions can also be found. In social science concept, there are two driver which linked to coral reefs conservation effort: proximate driver and distal driver (Fig. 1) [9]. However, frequently efforts are made to only focus on and include proximal driving factors, such as fishing restrictions [10]. Whereas the ultimate thing in coral reef conservation efforts is to overcome distal social drivers such as human behaviour. Therefore, the analysis of behaviour change can be an important indicator in conservation efforts of coral bleaching.

As show in Fig. 1, distal drivers are components in social systems that indirectly affect how people interact with coral reefs. Proximal drivers directly affect coral reef ecosystems (center). Coral reefs provide various important ecosystem benefits for humans, thus affecting aspects of human well-being. The one-way arrow shows the path from distal drivers to human well-being. The two-way arrows show the complex interrelationships and reciprocity that occur between the various components.

Automatic classification of behaviour stages has been carried out [11]on the topic of energy use as a campaign effort on climate change. Several machine learning models were used: Naive Bayes, Support Vector Machine (SVM), and Decision Tree, which were trained for the 5-stage behaviour change classification task. Data obtained from Twitter with topics: *Earth Hours 2015* (EH15), *Earth Hours 2016* (EH16), dan *Conference of the Parties* 21 (COP21). This study concludes that most users are in the Desirability stage and in the second position is the Can Do stage. This shows that in the climate change campaign, some people already have concerns and desires to change their behaviour and some have taken action.

The use of transformer-based deep learning models that are trained specifically on certain types of text and on certain topics shows better performance than models trained in general with conventional texts. In one of the Tweet classification competitions about Covid-19, the top 3 rankings were occupied by teams using the COVID-Twitter-BERT (CT-BERT). This model is based on the BERT-Large model, but has been further trained with 22.5 million Covid-19 related Tweets [12]. NutCracker Team [13], first place, collaborated the CT-BERT model with RoBERTa using a two-level ensemble. Whereas NLP North Team [14], second place, use stand-alone CT-BERT model dan UIT-HSE Team [15], third place, ensemble several CT-BERT models that have different hyperparameters with soft voting and hard voting techniques.

One of the transformer-based pre-trained deep learning models that are specially trained on the type of English Tweet text is BERTweet [16]. This model is designed to address the challenges of characteristic differences between Tweets and conventional texts such as Wikipedia and news articles. Tweets tend to be shorter and use informal vocabulary and abbreviations. Thus, BERTweet model is specially trained with 850 million English Tweets. This model has outperformed its competitors, RoBERTa [17] and XLM-R [18], on various tasks such as POS-Tagging, NER, and Text Classification, across various datasets.

This study utilize deep learning model, BERTweet, which has been specifically trained on Tweets and proven to overcome other strong baseline models [16], to build a 5 stages classification system for behaviour change on the topic of coral bleaching. The data is obtained from Twitter at a certain time in 2021. There are two proposed design that will be used: embedding extraction which utilizes the output of each encoder layer in BERTweet and stacking ensemble which uses several BERTweet models with different hyperparameters that are ensembled using a logistic regression model. Previous study reported that embedding extraction approach is a good transformer-based task-specific model for a transformer encoder and only need one run of transformer training to create various extraction scenarios so it is cost effective in computing resources [19]. The ensemble technique is proven to provide an increase in performance because deep learning ensemble models derive the advantages of the deep learning model itself and the advantage of the ensemble learning [20]. The main benefit of making this model is the fast and vast automation in analyzing the stage of behaviour change towards the coral bleaching campaign which is so limited compared other environmental issue.

The main novelty carryout in this experiment are creating deep-learning-based model for classification 5 stages of behaviour change on coral bleaching topic and new exploration on hyperparameter configuration and logistic regression model selection in stacking ensemble design. Through this experiment, all proposed modification design proven to outclass all baseline and original model.

## Related works

### The value of coral reefs and its threats

Coral reefs provide food and habitat for marine species, like small fishes and generate structure barrier on coastline to protect bioerosion and physical erosion [21]. There are also many benefits from coral reefs for humans, which are fisheries, coastal protection, medicine and tourism [22]. Coral reefs contribute as a source of protein for many organisms and a source of local income, so it cannot be separated from the coastal ecosystem. Coral reefs are also the source of the success of reef tourism, due to its economic value and on-reefs activities, such as diving, snorkeling, glass-bottom boating and tourism attractions, such as seafood and scenery [23]. Oceans produce about half of the oxygen in the earth and absorbing about 30 percent of carbon dioxide. Coral reefs are the foundation of the ocean health and without them, marine life would not exist [24].

In the midst of its crucial existence for many organisms, coral reefs experienced various threats. It is reported that mass bleaching events occurred around the world in 1998, 2002, 2010, and 2016 along with individual coral bleaching happens more often [25]. During the 2016 mass bleaching event in the Great Barrier Reef (GBR), only 8.9% of reefs survived, compared to the last two mass bleaching events, 42.4% of reefs survived in 2002 and 44.7% survived in 1998 [1]. Coral bleaching also occurred in Maldives in 2016, leaving only less than 6% of the total coral population surviving [26].

### Computer science and coral reef conservation

There are several attempts to mitigate coral bleaching that are related to computer science. The detection of coral species with the Artificial Neural Network [27] was built by collecting several images from the West Atlantic Ocean, Eastern Australia, Central Indian Ocean, Southeast Asia and Central Pacific Ocean, then used as training and testing dataset. There is also an attempt to save corals by classifying coral scenery images in the Gulf of Eilat to see if they are urchin, healthy corals, or dead corals based on the image recognition using Convolutional Neural Network [28].

### Social media data in conservation

Conventional extensive and large data collection will take a lot of money, time, and not even have sufficient resources available but social media which has been increasing over years can become an alternative. However, the use of social media data in conservation science is very limited and only available in a few sectors. In the conservation area, social media data not only can be used to raise awareness, but also to assess the attention received by particular species or ecosystems on social media platforms. Data from social media could give a direct behavioural basis for assessing public participation in biodiversity conservation. Temporal studies of social media data might also be utilized to better understand changes in biodiversity preference across time [7].

Researchers are using social media data for conservation science by gathering information from user's profile from a certain social media [29]. Flickr posts and Twitter tweets are used for assessing global popularity and threats to Important Bird and Biodiversity Areas (IBAs) by calculating the density of social media posts from geographical location worldwide ranging between February 2016 and June 2017 [30]. Instagram posts also contributes to data source for Hawaiian Monk Seal conservation by filtering post with hashtag '#monkseal' and check if the photo contains human disturbance or not by looking at Human-Wildlife interaction rule [31]. Sogou and WeChat posts also be used to strengthen public awareness of wildlife conservation in China by classifying them to six categories and also analyze differences among data groups using Kruskal—Wallis Test [32]. Twitter tweets can also be used to monitor five stages of behaviour changes according to Five Doors Theory, so furthermore to understand targeted strategies and intervention for driving intended change that are associated with climate change [33].

### The power of BERT modification

In particular, the development of deep learning for text classification has made extensive use of Google’s BERT. In a tweet classification task about COVID-19, which classifies whether a tweet is an informative tweet or not, various BERT models that have been modified and specifically trained are used, such as: BERT + [34], CT-BERT [14], and BERTweet [16]. Top result in the tweet classification task about COVID-19 was achieved by CT-BERT model and its modification. This is because that model is a BERT model who was specially trained on Tweets and on the topic of COVID-19.

### Five doors theory of behavior change

Robinson introduced a theory called the Five Doors Theory which focuses more on enabling the relationship between human behaviour and modifying technological and social contexts [8]. Five Doors Theory consists of 5 stages:

- Desirability: People in this stage are motivated to reduce their frustration, which can be daily discomfort or about deeper personal frustration or sadness or wanting something to change for the better.
- Enabling context: People in this stage are changing their environment to allow for new behaviours. This includes infrastructure, services, social norms, governance, knowledge—literally anything that can have a positive or negative influence on certain behaviours, but they are only planning what they can do to change their environment, not to the point of acting.
- Can do: People in this stage are already acting and doing something to change their environment. People at this stage also give suggestions for taking action to contribute to their environment.
- Buzz: People in this stage share their happy experiences and success stories.
- Invitation: People in this stage invite and involve others for a specific purpose.

Each stage in Five Doors Theory has its own linguistic pattern. According to [11], the linguistic pattern of the Desirability stage usually expresses negative sentiments and emotions such as frustration, anger, and personal sadness. This stage usually includes a URL to reveal the fact, and a question asking for help on how to solve the problem/frustration they are facing. The linguistic pattern of the Enabling Context stage is usually expressed in neutral sentiments and emotions. This stage generally provides facts on how to solve a problem based on facts, accompanied by a URL and conditional to show that, by taking a particular action, benefits are potentially obtained. The linguistic patterns of the Can Do stages are usually expressed with neutral sentiments and generally contain suggestions and commands aimed at oneself and others. The linguistic pattern of the Buzz stage usually has positive sentiments and emotions of happiness and joy, as the tweets generally talk about users' success stories and about the actions that they have taken in their engagement with climate change and sustainability. The linguistic pattern of the Invitation stage usually has positive sentiments and happy emotions, as it focuses on engaging others in a positive way. The text generally contains vocative forms that call on others to join this movement. The example sentences for each stage of the five stages of behaviour change can be seen in Table 1.

**Table 1:** Tweet example according to Five Doors Theory

Stage	Tweet example
Desirability	Our buildings need 40% of all energy consumed in Switzerland!
Enabling Context	I am considering walking or using public transport at least once a week
Can Do	If you are not using it, turn it off!
Buzz	I’m so proud when I remember to save energy and I know however small it’s helping
Invitation	Take 15 min out to think about what you do now and what you could do in the future. Read up on the subject and decide what our legacy will be

Five Doors Theory is being used in many projects, such as making a conceptual design to raise collective awareness and leverage energy savings by adapting and applying Five Doors Theory into the platform design [35]. Five Doors Theory is also being used as a base for the Climate Change Multitask Game [36]. There are several features that are used in the game, which are number of pledges answered by the user, ratio of pledges the user is already doing, ratio of pledges accepted, ratio of pledges refused, number of points per visit and social logging. Five Doors Theory can also be used to reflect behavioural stages in Tweets. The tweets will be extracted and categorized into each class by its linguistic pattern with GATE. The features that are extracted are polarity, emotions, directives or if the tweet consists of URL or not. The tweets will be tested on three models, which are Naive Bayes, Support Vector Machine and J48. The J48 model has the best performance because it has the highest accuracy, precision, recall and F1 score [11].

## Methodology

In 1960s until 2010, statistic-based or machine learning text classification models were ruled, such as Naïve Bayes (NB), K-Nearest Neighbor, and Support Vector Machine (SVM). These models need features engineering effort which costly and time-consuming. Furthermore, these models usually neglect the sequential structure or contextual information in text data, so make it challenging to understand the text semantic information. Nowdays, the text classification start to shift into deep learning, such as transformer-based models, which keep off designing rules and features by humans and also automatically provide semantically meaningful representations [37].

The use of transformer-based models [38] has now become a trend in NLP tasks, including text classification tasks. Models trained specifically for certain text types can outperform models trained with conventional text types. In this study, the text is in the form of tweets, so the main model explored in this study is the BERTweet model. BERTweet has the same architecture as the BERT base, trained with RoBERTa pre-training procedures, and specially trained on 850 M English Tweets. The different characteristic between Tweet and conventional text, Tweet tend to be shorter and use informal vocabulary and abbreviations, become the reason to choose text-specific trained model.

**Fig. 2:**
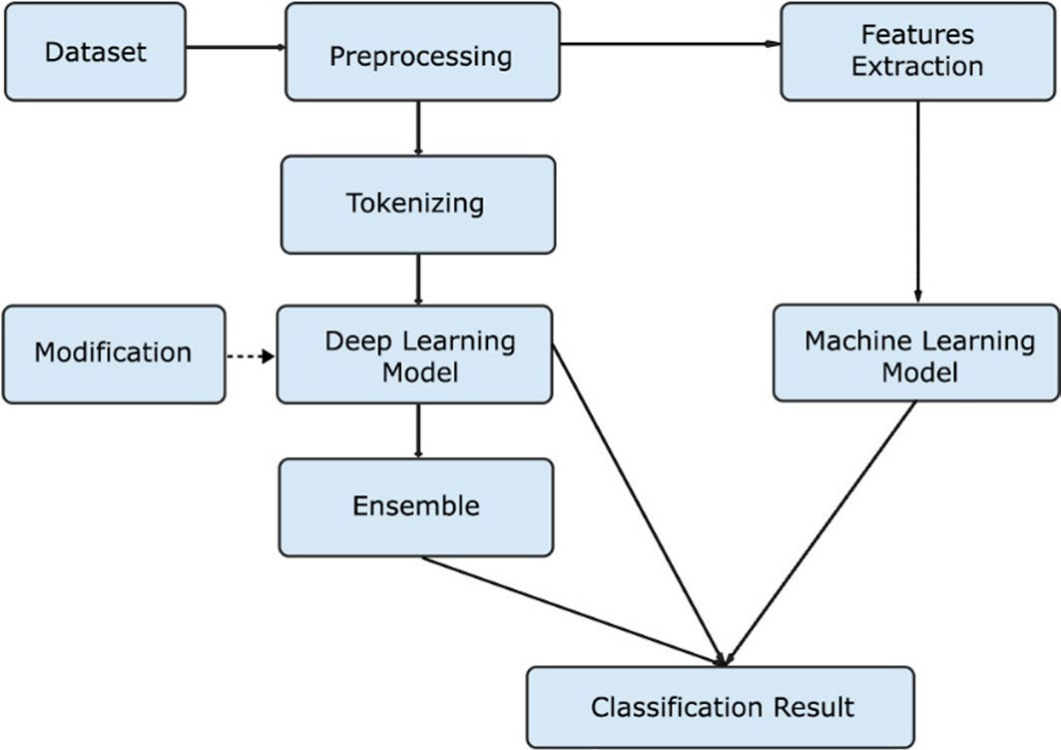
General experiment flow

In this study, the outline of the experimental flow that will be carried out can be seen in Fig. 2. There are several machine learning models as the baseline model and several deep learning models as the baseline and the main design of the proposed designs. There are 2 main designs of the proposed model: BERTweet embedding extraction and BERTweet stacking ensemble.

To evaluate the classification performance of the designed models, 2 metrics are used: accuracy and F1 score. To calculate accuracy and F1 score, the value of precision and recall must also be calculated. Precision is the ratio of correct positive predictions from total positive predictions. Recall is the ratio of correct positive prediction from data that are actually positive.

To calculate those metrics, defined for any classifier *f*: *D → C* = {1, …, *n*} and finite set *S ⊆ D* × *C*, let *m*^*f,S*^ ∈ ${\mathrm{N}}_{0}^{n\times n}$ be a confusion matrix, where =${m}_{ij}^{f, S}$|*{s ∈ S | f(s*_*1*_*)* = *i ∧ s*_*2*_ = *j*}|. For any such matrix, let *P*_*i*_, *R*_*i*_ and *F1*_*i*_ denote precision, recall and F1-score with respect to class *i*:

1 $${P}_{i}= \frac{{m}_{ii}}{\sum_{x=1}^{n}{m}_{ix}} ; {R}_{i}= \frac{{m}_{ii}}{\sum_{x=1}^{n}{m}_{xi}} ;F{1}_{i}=H\left({P}_{i}, {R}_{i}\right)= \frac{2{P}_{i}{R}_{i}}{{P}_{i}+{R}_{i}}$$

with *P*_*i*_, *R*_*i*_, *F1*_*i*_ = 0 when the denominator is zero. Precision and recall are also known as positive predictive value and sensitivity.

For every scenario, the F1 score are computed using Macro F1 which follow the step of computing the F1 score for each class and then averaging it via arithmetic mean, the mathematical formula can be seen in:

2 $$F1= \frac{1}{n}\sum_{x}F{1}_{x}=\frac{1}{n}\sum_{x}\frac{2{P}_{x}{R}_{x}}{{P}_{x}+{R}_{x}}$$

### Baseline

The task of classifying behaviour changes in the phenomenon of coral bleaching is a new and less popular task, therefore no previous research has been found that can be used as a reference for model performance. Simple yet reasonable models are chosen for baseline model. From the deep learning approach, the BERT-large model was chosen which was not specifically trained on Tweet-type text, while the machine learning approach used 4 classifiers: Support Vector Machine (SVM), Logistic Regression (LR), K-Nearest Neighbors (KNN), and Random Forest (RF), with features obtained using the word embedding Glove pre-trained Twitter-100.

For the SVM, LR, KNN, and RF models, the input data is in the form of tweets whose word representation is extracted using pre-trained Global Vectors for Word Representation (Glove) Twitter-100, then the vectors of each word in a tweet are summed and averaged. The BERT model receives input in the form of tweets that have been cleaned and tokenized with the BERT tokenizer.

### Dataset

**Fig. 3:**
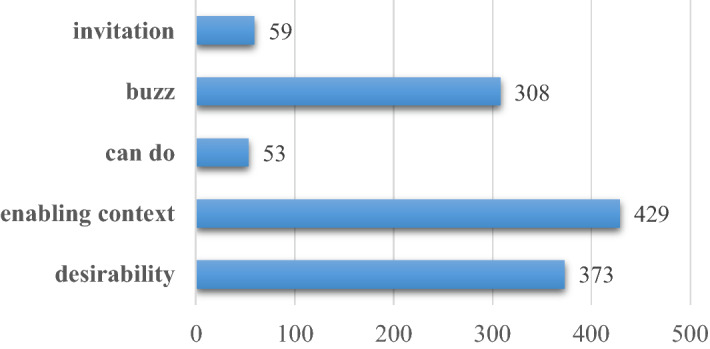
Distribution of each class on dataset

The dataset was taken from a trusted repository, Mendeley Data, with title "An Annotated Dataset for Identifying Behaviour Change Based on Five Doors Theory Under Coral Bleaching Phenomenon on Twitter" [39]. This dataset contains 1,222 tweets with keywords related to coral bleaching that have been annotated according to the behaviour change stages. The distribution of data for each class of behaviour change can be seen in Figure 3. The distribution of Can Do and Invitation class are uneven, much less than other classes.

The dataset was split into training and testing set. The splitting process is carried out by maintaining the ratio of the distribution of data in each class with proportion of 80% for training set and 20% for testing set. The distribution of classes in the training and testing sets can be seen in the Table 2.

**Table 2:** Distribution of each class on training and testing set

Set	Number of Tweets					
	Desirability	Enabling context	Can Do	Buzz	Invitation	Total
Training	298	343	43	246	47	977
Testing	75	86	10	62	12	245

### Pre-processing

**Fig. 4:**
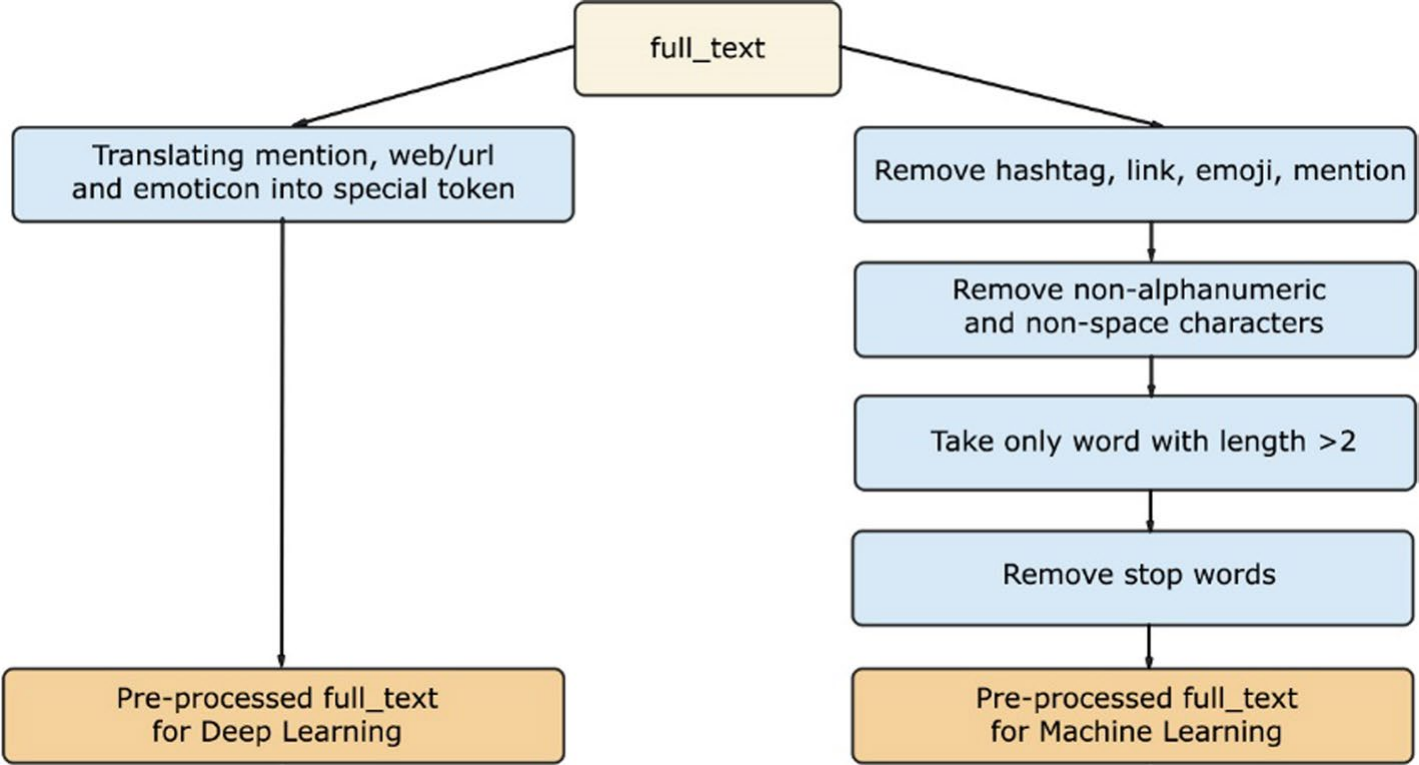
Preprocessing path

To accommodate the two types of models used in this research, deep learning models and machine learning models, there are 2 main streams of pre-processing applied to tweets, which can be seen in Figure 4. Each of the results of the pre-processing will then be used as material for feature extraction for machine learning models and tokenization for deep learning models.

**Fig. 5:**
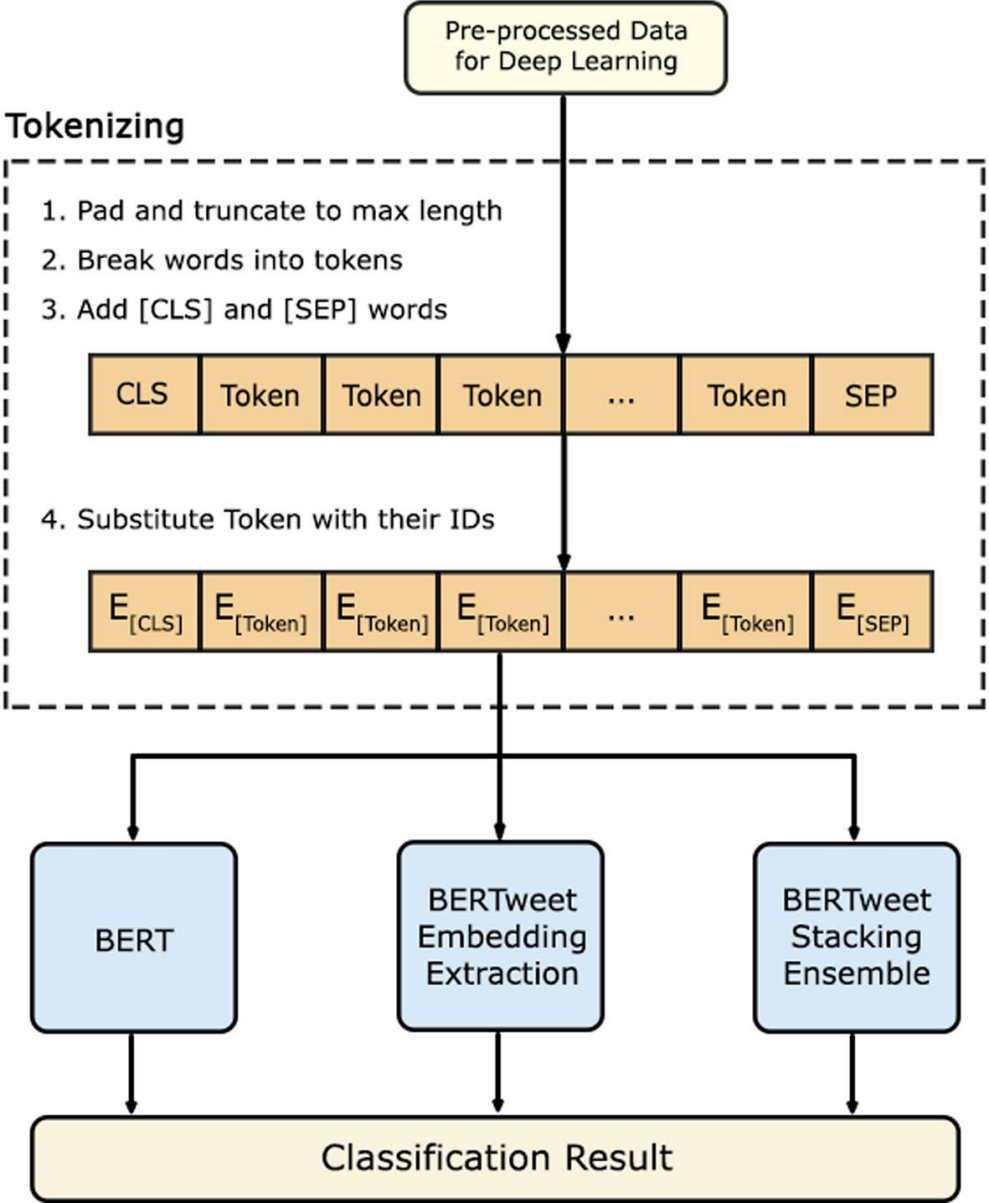
Deep Learning tokenization procedures

More detail about tokenization procedure for deep learning experiment can be seen in Figure 5. In general, there were 3 types of experiment using deep learning architecture: BERT as baseline model for deep learning, BERTweet Embedding Extraction and BERTweet Stacking Ensemble as enhanced methods to overcome classification task on this research.

### BERTweet embedding extraction

Each Transformer layer within the BERTweet model learns different and unique information. Several experiments using the BERT model have been done using fine-tuning approaches such as BERT Large and BERT Base. However, it is reported that embedding extraction approach, where embedding results from each encoder extracted as features, has certain advantages, such as being a good transformer-based task-specific model for a Transformer encoder because not all tasks can be easily represented by a default Transformer encoder architecture and getting results from many scenarios just by running the transformer encoder once and make cheaper models on top of it. From the result of the experiment, the performance of embedding extraction approach by concatenating the last four layers can match the performance of fine-tuning approaches such as BERT-base and BERT-large [19].

**Fig. 6:**
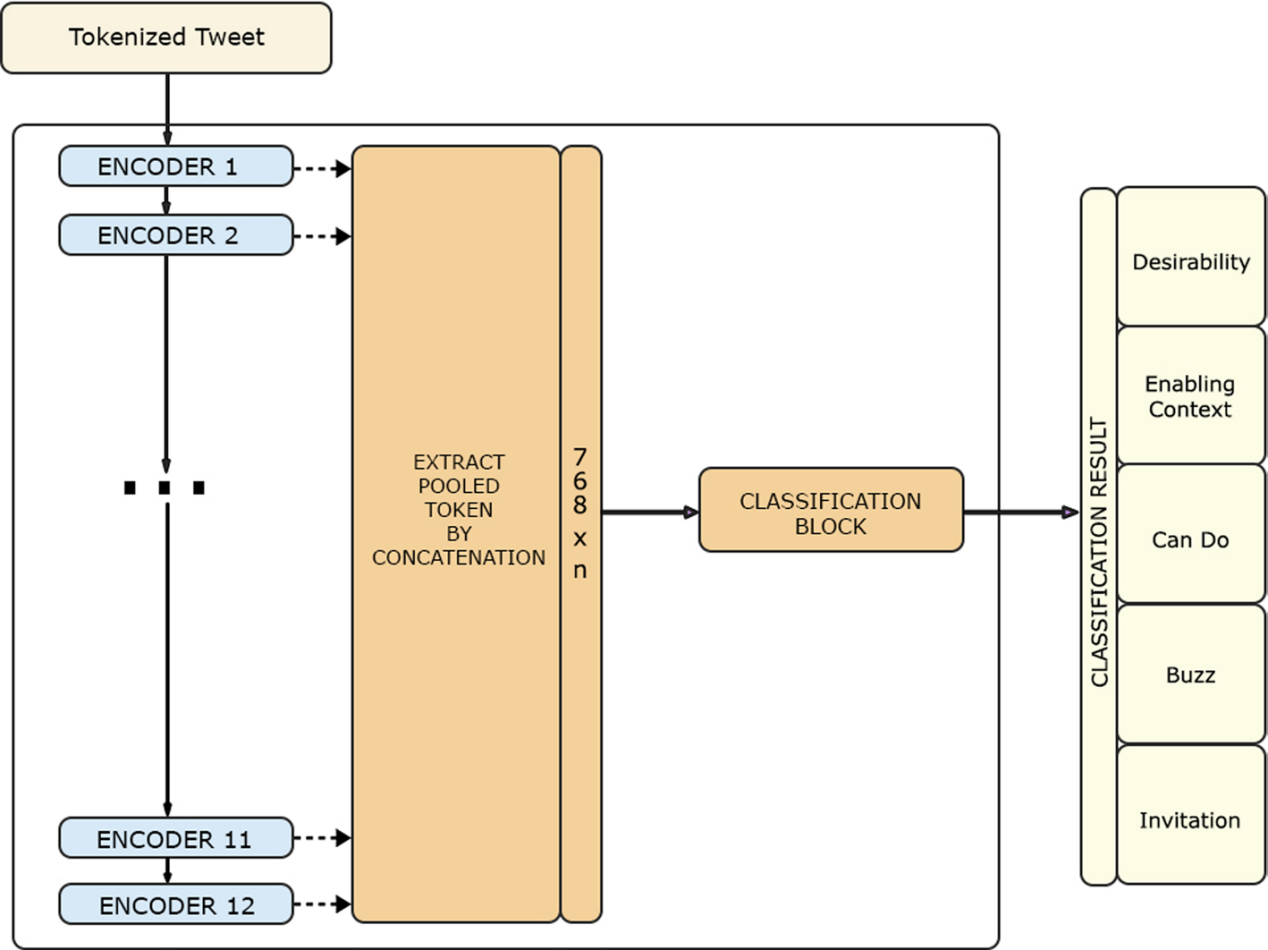
BERTweet Embedding Extraction configuration

The experiment is done by feeding the tokenized input to the Transformer block. There are 8 different scenarios of the extraction (Table 3). A Transformer block consists of 12 encoder blocks, but the extracted encoder blocks result depend on the setting that is used. In general, the flow of this experiment can be seen in Fig. 6. Each encoder block generates a CLS token embedding result (*e*_*1*_*, e*_*2*_*, e*_*3*_*, …, e*_*12*_) and those result are concatenated based on several combinations according to the scenarios to become input for classification block (*h*).

$${\text{h}}\, = \,concat(e_{c1,} e_{c2} , \, e_{c3} , \, \ldots , \, e_{cn} | \, c\, = \,\{ {\text{chosen encoder}}\} )$$

**Table 3:** BERTweet Embedding Extraction scenarios description

Scenario	Extracted Embedding
All 12 layers	*e*_*1*_ – *e*_*12*_
Last layer	*e*_*12*_
Last 4 layers	*e*_*9*_ – *e*_*12*_
Last 2 layers	*e*_*11*_ – *e*_*12*_
First 2 + Last 2	*e*_*1*_ – *e*_*2*_; *e*_*11*_ – *e*_*12*_
First + Last	*e*_*1*_; *e*_*12*_
Last 2 + Mid 2	*e*_*11*_ – *e*_*12*_; *e*_*6*_ – *e*_*7*_
Last + Mid	*e*_*12*_; *e*_*6*_

The classification block consists of dense layers and dropout layers to extract the concatenated pooled token and produce only 5 features at the end because there are 5 classes in this classification task. The initial hyperparameters in the experiments are learning rate and epsilon of 1e-4 in 7 epochs.

### BERTweet stacking ensemble

Minor differences in hyperparameter configurations can give different performance results for each model. Calibrating hyperparameters is key to increasing model performance in deep learning and NLP. Once adapted across methods, hyperparameter tuning significantly improves performance in every task. In many cases, modifying the setup of a single hyperparameter yields a larger increase in performance than shifting to a better algorithm or training on a larger corpus [40].

On this occasion, experiments were conducted with the difference in determining the value of learning rate and epsilon. The range of values to consider for the learning rate is less than 1.0 and greater than 1e-6, but these should not be taken as strict ranges and greatly depend on the parametrization of the model [41]. In a study [42], a lower learning rate, such as 2e−5, is necessary to make BERT overcome the catastrophic forgetting problem and an aggressive learn rate of 4e−4, the training set fails to converge. The epsilon is to avoid divide by zero error while updating the variable when the gradient is almost zero. So, ideally epsilon should be a small value, but a very small value will make normalization in weight update to 1. The trade-off is that the bigger epsilon, the smaller the weight updates are and thus slower the training progress will be.

After few initial tries, there are 2 values of learning rate and 2 values of epsilon which considered as combination choice for model configuration. Thus, there are 4 combinations of hyperparameter setting which can be seen in Table 4. All models (modelSE#1 to modelSE#4) are standard BERTweet model with modification on learning rate and epsilon. Those models will be used as the standalone model which will then be combined for stacking ensemble scenarios. In these experiments, the batch size was set as 4. Smaller batch size has an advantage over larger ones. Smaller batch size works better due to the trade-off between number of samples and number of updates [43]. This time the dataset used is not large, so it is possible to have a small batch size with the available computing resources. Thus, the weight update process will run more frequently, so significantly increasing training stability.

**Table 4:** The model configuration of the four individual models with batch size 4 and 9 epochs

Model	Learning Rate (lr)	Epsilon (eps)
modelSE#1	1.00E−05	1.00E−08
modelSE#2	1.00E−05	1.00E−12
modelSE#3	2.00E−05	1.00E−08
modelSE#4	2.00E−05	1.00E−12

*SE* stacking ensemble

**Fig. 7:**
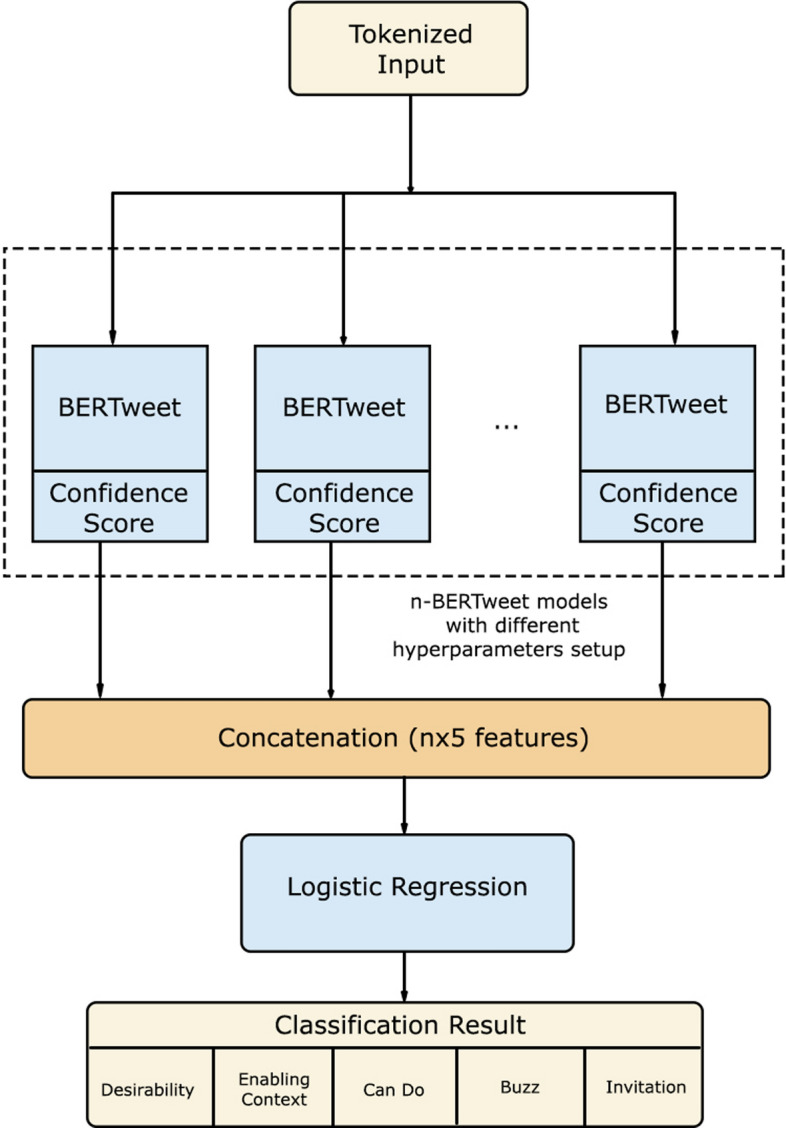
BERTweet Stacking Ensemble configuration

In Fig. 7, there are *n* BERTweet models which have different hyperparameters setup that will be ensembled using a stacking technique by treating the confidence score results from each BERTweet model (*b*_*1*_*, b*_*2*_*, b*_*3*_*, … , b*_*n*_) as input for a machine learning model (*h*) by concatenation.

$${\text{h}}\, = \,concat(b_{c1,} b_{c2} , \, b_{c3} , \, \ldots , \, b_{cn} | \, c\, = \,\{ {\text{chosen model}}\} )$$

Based on the previous studies shown that ensemble approach was effective [15], so the experiments have been carried out by combining all combination of 2 models from a total of 4 standalone models and combining all 4 standalone models. There are 6 scenarios of combination of 2 models which use *n* = 2 and a scenario of combination of 4 models which use *n* = 4. The combining technique is done using stacking technique, where each model is trained in parallel, then the results of confidence score for each class of each model are combined by concatenation, and in the end a machine learning model, logistic regression with SGD training, is used to provide the final prediction results. Each model produces 5 value of confidence score. The experiments of combining all combination of 2 models feed the machine learning model with 10 number of features and the experiment for combining all 4 models feed machine learning model with 20 number of features. Logistic regression model was selected because it is simple, fast, and computationally inexpensive [44]. Computational of deep learning models have cost quite expensive, therefore machine learning model with low cost is chosen.

For each combination experiment performed has its own logistic regression model which is partially trained for each epoch. So, the learning process of each logistic regression model is continuous every epoch. This allows for improved logistic regression model performance as the epoch progresses.

## Result

### Baseline

The three baseline models achieve accuracy in fifties to sixties and F1 scores in thirties to fifties. The results in Table 5 show that deep learning approach (BERT) that is not trained specifically for Tweet can still be defeated by the results of a machine learning model that derives features from Tweet-specific word embedding (SVM). All machine learning classifiers show results that are still quite low, this is a trade-off between accuracy and efficiency, where training machine learning models is not as costly as deep learning models.

**Table 5:** Baseline model results

Model	Accuracy	F1 score
BERT	0.6393	0.5692
SVM	0.6612	0.5230
Logistic regression	0.6122	0.5318
K-nearest neighbors	0.5755	0.4154
Random forest	0.5959	0.3742

### BERTweet embedding extraction

Each layer holds unique and different information, but they still can produce good results (above 0.75 accuracy and above 0.7 F1 score) which can be seen on Table 6. Standard BERTweet model (Last Layer) already has good results (0.7714 accuracy and 0.7298 F1 score) but with the extraction of the last 4 layers, the performance result on F1 score can be boosted. The most interesting part of this experiment is there are six scenarios of which have F1 score lower than its accuracy, there is a scenario of which has F1 score little bit higher to its accuracy and there is a scenario of which has F1 score far higher than its accuracy. In the scenario EE#3 and scenario EE#7, both classes have a much higher recall score in Can Do classes (around 0.8) than the other scenarios. The different extraction combinations of information that are held inside each layer of BERTweet are able to cause enhancement in model performance to classify classes that have a small amount of data. In this experiment, all scenarios predicted Invitation class very well and produced very high precision and recall for Invitation class (over 75%). Meanwhile, the best scenario in the experiment has decent precision and recall on Enabling Context class, only scoring 0.7073 for precision and 0.6744 for recall so it gives a decent F1 score for Enabling Context (0.6905). The scenario EE#3 has a fairly higher F1 score in each class than the scenario EE#7, so the average of all F1 score of the scenario EE#3 has higher F1 score than the scenario EE#7.

**Table 6:** BERTweet embedding extraction (EE) result with lr = 1e-4 and eps = 1e-4

Scenario	Description	Maximum accuracy	Maximum F1 score
**EE#1**	**Last Layer**	**0.7714**	**0.7298**
EE#2	All 12 Layers	0.7633	0.7406
**EE#3**	**Last 4 Layers**	**0.7673**	**0.7833**
EE#4	Last 2 Layers	0.7510	0.7484
EE#5	First 2 + Last 2	0.7510	0.7445
EE#6	First + Last	0.7673	0.7388
EE#7	Last 2 + Mid 2	0.7510	0.7589
EE#8	Last + Mid	0.7673	0.7214

Scenario EE#1 is bolded because it has the highest accuracy while scenario EE#3 is bolded because it has the highest F1 score

### BERTweet stacking ensemble

Every model obtains different performance results with a fairly obvious distance, which can be seen in Table 7. There are random factors, such as the initialization of weights, which also affect the results of the model's performance. However, by looking at the trend of several experiments that have been tried, the determination of the learning rate plays a major role in achieving the best results. In many trials, the highest performance result of one learning rate determination never reaches or exceeds the highest performance result of another learning rate determination value.

**Table 7:** BERTweet stacking ensemble (SE) result

Scenario	Description	Accuracy	F1 score
SE#1	modelSE#1	0.7429	0.6786
SE#2	modelSE#2	0.7592	0.7399
**SE#3**	**modelSE#3**	**0.7673**	**0.7473**
SE#4	modelSE#4	0.7429	0.7436
SE#5	combination1 (modelSE#1 and modelSE#2)	0.7755	0.7677
SE#6	combination2 (modelSE#1 and modelSE#3)	0.7551	0.7530
SE#7	combination3 (modelSE#1 and modelSE#4)	0.7551	0.7298
SE#8	combination4 (modelSE#2 and modelSE#3)	0.7388	0.7236
**SE#9**	**combination5 (modelSE#2 and modelSE#4)**	**0.7633**	**0.7829**
SE#10	combination6 (modelSE#3 and modelSE#4)	0.7592	0.7754
**SE#11**	**combinationAll (modelSE#1—modelSE#4)**	**0.7796**	**0.7945**

Scenario SE#3 is bolded because it has the best performance out of all standalone models, scenario SE#9 is bolded because it has the best performance out of the combination of two models and SE#11 is bolded because it has the best performance out of the combination of four models

The results of the performance of each model, the combination of 2 models, and the combination of all models can be seen in able 6. An accuracy of 0.7796 and an F1 score of 0.7945 were obtained as the best results on the test dataset for the classification of 5 classes of behaviour change stages. The combination of all models gives the best accuracy and F1 score results compared to all the results of standalone model and other combinations. For the standalone model, the best result was obtained by modelSE#3 with 0.7673 on accuracy and 0.7473 on F1 score. In general, the value of accuracy and F1 score has increased by performing this ensemble of stacking techniques, both in the combination of 2 models and the combination of all models. In stacking combination of 2 models, all combination except scenario SE#8 resulted in improved performance on either one or both metric (accuracy and F1 score).

The combination of modelSE#2 and modelSE#3 (scenario SE#8) has lower performance than the two standalone model which combined. This can be happened because the machine learning block can produce prediction which are never generated by two standalone model. Scenario SE#8 has the highest the number of machine learning block prediction which are never generated by two standalone model which is 7 and only 1 matched the original label. Even though modelSE#2 and modelSE#3 are the two models with best performance. In the combination of all models, there are no machine learning prediction that never proposed by all standalone models.

Among the differences in the predictions proposed by each model, machine learning blocks can help provide correct predictions up to 50 s to 60 s percent of the total cases where the predictions proposed by each model are different, except for scenario SE#8 which is only about 37 percent. The stacking ensemble technique using machine learning blocks is able to correct several wrong predictions in each model. Scenario EE#11 is the combination that most helps correct wrong predictions, for modelSE#1 there are 22 predictions that have been successfully corrected, for modelSE#2 there are 18, for modelSE#3 there are 6, and for modelSE#4 there are 16.

There are tweets that are quite difficult and confusing to classify between the 2 classes. Based on the statistics of the combination of all models, the four models can propose different predictive results and the differences that are most often found are proposing Enabling Context and Desirability with 32 cases, followed by Enabling Context and Buzz with 15 cases, and for the others only under 10 cases. The Desirability and Enabling Context classes both have characteristics supported by facts. Therefore, these two classes are two classes that are rather difficult to predict by the model. Some models predict as Enabling Context, but some other models propose Desirability predictions. In addition, the presence of a certain language style to attract reader’s attention makes Enabling Context and Buzz classes also a challenge, for example, it can be seen in Table 8, the tweet should be included in the Buzz class because it tells the story of a successful coral restoration effort, but because there is a language style that uses conditional sentences that is one of the linguistic characteristics of the Enabling Context class, so most models propose Enabling Context predictions.

**Table 8:** Example of Buzz tweet which predicted as other class. Label 0 stand for Desirability, 1 stand for enabling context, and 3 stand for Buzz

Tweet	Actual Label	modelSE#1 prediction	modelSE#2 prediction	modelSE#3 prediction	modelSE#4 prediction
Think these corals are bleached? Think again! If this coral were bleached, we would see the entire colony slowly lose its color in a process called “paling.” Those white tips you see are actually new growth! : JD Reinbott/Coral Restoration Foundation™ https://t.co/a32M4ZaM1x	3	1	1	1	0

### Discussion

Of the two main model designs, the two scenarios that have the best performance are the scenario EE#3 for the BERTweet embedding extraction design and the scenario SE#11 for the BERTweet stacking ensemble design. Both scenarios achieve an macro F1 score of more than 78 percent. Obtaining precision, recall, and F1 score values ​​for each class and their average for the scenario EE#3 can be seen in Table 9 and for the scenario SE#11 it can be seen in Table 10.

**Table 9:** Results for BERTweet embedding extraction scenario EE#3

	Precision	Recall	F1 score
Desirability	0.7349	0.8133	0.7722
Enabling Context	0.7073	0.6744	0.6905
Can Do	0.6667	0.8000	0.7273
Buzz	0.8947	0.8226	0.8571
Invitation	0.9091	0.8333	0.8696
Average	0.78255	0.788733	0.783322

**Table 10:** Results for BERTweet stacking ensemble scenario SE#11

	Precision	Recall	F1 score
Desirability	0.8261	0.7600	0.7917
Enabling Context	0.7000	0.7326	0.7159
Can Do	0.6429	0.9000	0.7500
Buzz	0.8525	0.8387	0.8455
Invitation	0.9091	0.8333	0.8696
Average	0.7861	0.8129	0.7945

In general, the scenario SE#11 is the best design in terms of the highest accuracy, precision, recall, and F1 score values ​​than all other scenarios. The precision, recall, and F1 score values ​​in each class are also fairly stable in the good category.

#### Dealing with unbalanced data

Although the distribution of the amount of data in each class is not balanced, the existing model design can give good results, even in classes with very little data. The Can Do and Invitation classes are the classes with the least data, each only about 4 percent of the total data. However, the model's performance for classifying Invitation class is very good. The sensitivity (recall) of both models to tweets which are the Invitation class is very high, up to 83.3 percent. The precision achieved by both models in the Invitation class is also very high, up to 90.9 percent. This shows that both models have recognized the characteristics of the Invitation class well, although there are not many examples of data.

There is a bit difference in the classification performance results in the Can Do class. Both models are quite sensitive to tweets that are in the Can Do class, seen from the recall value of 80 percent in the scenario EE#3 and 90 percent in the scenario SE#11. However, the precision of the two models for classifying the Can Do class is still relatively low. Of all the Can Do predictions, only about 60 percent are actually Can Do classes. With less data and a wider variety of tweets, the Can Do class is more difficult to classify precisely by the two models. Many of Can Do's predictions are not true, indicating that this class has characteristics that overlap or are similar to other classes.

#### Facing bias on data characteristics

If it is seen from the confusion matrix in Table 11 for the scenario EE#3 and Table 12 for the scenario SE#11, the data prediction error is not far from the actual behaviour change stage. For example, out of 75 Desirability tweets, only 13 tweets were predicted as Enabling Context and one tweet was predicted as Can Do. Enabling context is the stage of behaviour change right after Desirability. However, for Enabling context tweets, there are still quite a lot of data that are predictable at other stages that are quite far away, such as Invitation class. This can happen because indeed the tweets in the Enabling Context class have far more diverse variations than other classes. In the Enabling Context class, tweets can be in the form of news/knowledge/facts and suggestions for solutions to many aspects related to the topic of coral bleaching. Also known, that Desirability class tend to have fact/news to support the frustration, Can Do and Invitation tweet also tend to give suggestion, and Buzz tweet sometimes also have some knowledge to share.

**Table 11:** Confusion matrix BERTweet embedding extraction scenario EE#3

Actual	Predicted					
		Desirability	Enabling Context	Can Do	Buzz	Invitation
	Desirability	61	13	1	0	0
	Enabling Context	20	58	2	5	1
	Can Do	0	2	8	0	0
	Buzz	2	9	0	51	0
	Invitation	0	0	1	1	10

**Table 12:** Confusion matrix BERTweet stacking ensemble scenario SE#11

Actual	Predicted					
		Desirability	Enabling Context	Can Do	Buzz	Invitation
	Desirability	57	17	1	0	0
	Enabling Context	11	63	3	8	1
	Can Do	0	1	9	0	0
	Buzz	1	9	0	52	0
	Invitation	0	0	1	1	10

The model in the scenario EE#3 is still less sensitive to tweets in the Enabling Context class. Only about 67 percent of the total Enabling Context tweets can be recognized by the model. As for the other classes, this model is quite sensitive with an average recall above 80 percent for each class. On the other hand, the model in the scenario SE#11 is quite sensitive in every class, with the lowest recall of around 73 percent in the Enabling Context class.

#### Outperform baseline models

**Fig. 8:**
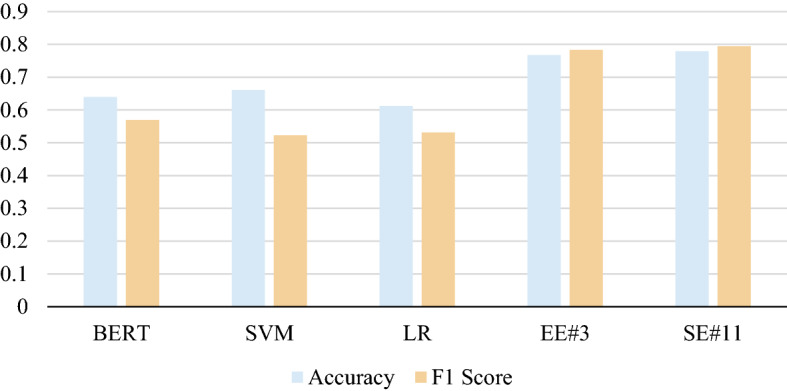
Performance comparison between baseline models and two best scenarios (EE#3 and EE#11)

When compared to the baseline, all scenarios obtained much better results, the performance comparation can be seen in Fig. 8. Three baseline models achieved accuracy around sixties and F1 score around fifties. In the other hand all scenarios proposed in this study achieved accuracy and F1 score around seventies. BERTweet, BERT model which is specially trained on certain types of text, outperforms BERT which is trained only with conventional text. Machine learning whose features are extracted with word embedding which is specially trained with tweet text also has not been able to outperform the BERTweet model.

## Conclusion

Monitoring behaviours change as an indicator of the success of coral bleaching campaigns requires new breakthroughs that cover a wider area and a faster time. The use of social media has been increasing over the years, including Twitter. Therefore, the idea arose to create a classification model that can analyze the stage of behaviour change based on tweets sent by users. By using the BERT model which is specially trained on the type of Tweet text, BERTweet, a reliable classification model has been successfully designed. The performance of BERTweet in general has outperformed the general BERT model and the Machine Learning model whose features are extracted by word embedding which is specially trained on the tweet text type.

In this study, the general BERTweet model was coupled with modified design using 2 techniques: embedding extraction and stacking ensemble. The two proposed techniques result in improved performance compared to the standalone BERTweet model. The experimental results of the embedding extraction technique are dominated by scenario EE#1 and scenario EE#3. The model in scenario EE#1 achieved an accuracy of 0.771, outperforming every scenario in embedding extraction technique, but scenario EE#3 achieved highest F1 score 0.7833 among all other scenarios. The experimental results of the stacking ensemble technique with the scenario SE#11 in general outperformed all other scenarios. The model in the scenario SE#11 achieved an accuracy of 0.7796 and an F1 score of 0.7945, this shows a good performance for classifying 5 classes of behaviour change stages. Even though there is an imbalance of data in certain classes, it only has around 4% of total data, but the existing model has a high sensitivity in each class.

The best proposed model achieved all of the precision, recall, and F1 score values for each class are above 70 percent, except for the Can Do class which has a precision of around 64 percent, but still gets an F1 score of 75 percent because the recall in this class is the highest (90 present). From this study, it is concluded that an automatic classification model for classifying the 5 stages of behaviour change based on Five Doors Theory on the Twitter platform can be made using a modified BERTweet and obtain satisfactory results. Hopefully with the automatic classification model that can be made, monitoring the success of the coral bleaching issue campaign can be carried out in a better way.

The focus of this study is to prove that BERTweet is suitable to be used for classifying Tweet into five stages of behaviour change on coral bleaching topic and also find out the best scenario of BERTweet modification which can handle and perform better than original BERTweet. Therefore, no extensive features engineering is done. Furthermore, deep learning model also does not need that because the main advantage using deep learning is the model ability to learn and create its needed features. However, in the further study, extensive features engineering on specific coral bleaching topic maybe done to help improve the performance and make more choices of development such as utilize machine learning model more aggressively.

## Data Availability

The datasets for this study are available on Mendeley Repository [39].

## Abbreviations

BERT: Bidirectional Encoder from Transformer
CLS: Classification
CNN: Convolutional Neural Network
COP21: Conference of the parties 21
CT-BERT: COVID-Twitter-BERT
EE: Embedding extraction
EH15: Earth hours 2015
EH16: Earth hours 2016
GATE: General Architecture for Text Engineering
GloVE: Global Vectors for Word Representation
GBR: Great Barrier Reef
IBA: Important Bird and Biodiversity Areas
NER: Named Entity Recognition
NLP: Natural language processing
POS: Part of Speech
RNN: Recurrent Neural Network
RoBERTa: Robustly Optimized BERT Pre-training Approach
SE: Stacking ensemble
SGD: Stochastic Gradient Descent
SVM: Support Vector Machine
URL: Uniform Resource Locator
XLM-R: Cross-Lingual Model – Roberta
